# Development of zebrafish medulloblastoma-like PNET model by TALEN-mediated somatic gene inactivation

**DOI:** 10.18632/oncotarget.19424

**Published:** 2017-07-21

**Authors:** Jaegal Shim, Jung-Hwa Choi, Moon-Hak Park, Hyena Kim, Jong Hwan Kim, Seon-Young Kim, Dongwan Hong, Sunshin Kim, Ji Eun Lee, Cheol-Hee Kim, Jeong-Soo Lee, Young-Ki Bae

**Affiliations:** ^1^ Comparative Biomedicine Research Branch, Research Institute, National Cancer Center, Goyang, Republic of Korea; ^2^ Department of Biology, Chungnam National University, Daejeon, Republic of Korea; ^3^ Genome Structure Research Center, Korea Research Institute of Bioscience and Biotechnology, Daejeon, Republic of Korea; ^4^ Clinical Genomic Analysis Branch, Research Institute, National Cancer Center, Goyang, Republic of Korea; ^5^ Precision Medicine Branch, Research Institute, National Cancer Center, Goyang, Republic of Korea; ^6^ Department of Health Sciences and Technology, Samsung Advanced Institute for Health Sciences and Technology, Sungkyunkwan University, Gangnam-gu, Seoul, Republic of Korea; ^7^ Disease Target Structure Research Center, Korea Research Institute of Bioscience and Biotechnology, Daejeon, Republic of Korea; ^8^ Convergence Research Center for Dementia DTC, Korea Institute of Science and Technology, Seoul, Republic of Korea; ^9^ Department of Functional Genomics, Korea University of Science and Technology, Daejeon, Republic of Korea; ^10^ Tumor Microenviroment Research Branch, Research Institute, National Cancer Center, Goyang, Republic of Korea

**Keywords:** medulloblastoma, PNET, MPNST, TALEN, somatic inactivation

## Abstract

Genetically engineered animal tumor models have traditionally been generated by the gain of single or multiple oncogenes or the loss of tumor suppressor genes; however, the development of live animal models has been difficult given that cancer phenotypes are generally induced by somatic mutation rather than by germline genetic inactivation. In this study, we developed somatically mutated tumor models using TALEN-mediated somatic gene inactivation of *cdkn2a/b* or *rb1* tumor suppressor genes in zebrafish. One-cell stage injection of *cdkn2a/b*-TALEN mRNA resulted in malignant peripheral nerve sheath tumors with high frequency (about 39%) and early onset (about 35 weeks of age) in F0 *tp53^e7/e7^* mutant zebrafish. Injection of *rb1*-TALEN mRNA also led to the formation of brain tumors at high frequency (58%, 31 weeks of age) in F0 *tp53^e7/e7^* mutant zebrafish. Analysis of each tumor induced by somatic inactivation showed that the targeted genes had bi-allelic mutations. Tumors induced by *rb1* somatic inactivation were characterized as medulloblastoma-like primitive neuroectodermal tumors based on incidence location, histopathological features, and immunohistochemical tests. In addition, 3′ mRNA Quanti-Seq analysis showed differential activation of genes involved in cell cycle, DNA replication, and protein synthesis; especially, genes involved in neuronal development were up-regulated.

## INTRODUCTION

Medulloblastoma (MB) is the most common type of malignant solid tumors in the pediatric brain, which accounts for 20% of all pediatric tumors. It occurs in the external granular layer (EGL) of the cerebellum and may undergo metastasis (reviewed in [1]). Based on recent transcriptional profiling, MB can be classified into four subgroups: WNT subgroup, Sonic Hedgehog (SHH) subgroup, Group 3, and Group 4 [2]. The WNT subgroup accounts for ˜10% of all MB and usually has good prognosis; this subgroup involves mutations in genes including *TP53*, *CTNNB1, AXIN1, SMARCA4*, and *CREBBP*. The SHH subgroup accounts for up to 30% of MB and contrarily shows poor prognosis. Loss of function mutations are found in genes *PTC1/2* and *SUFU* whereas gain of function mutations are found in *SMO* and *GLI1/2*; furthermore, Notch and PI3K signaling (IGFRII) are activated in the SHH subgroup. Group 3 accounts for ˜25% of all MB and carries no *TP53* mutations while showing poor prognosis, immature histopathology, and highly metastatic property. Group 4 accounts for ˜35% of all MB; similar to Group 3, Group 4 carries no *TP53* mutations while mutations are often identified in genes including *OTX2, N-MYC, FST*, and *CDK6*.

The first MB model was SHH receptor *Ptc1* heterozygote (+/−) mice which developed MB with low penetrance (< 7.4%) [3]. A conditional knockout mouse model for *Ctnnb1*, a critical component of Wnt signaling, was generated to reveal Zic1+ precursors in the dorsal brainstem as cells of origin of the WNT subtype MB [4]. In addition, a concomitant knockout mouse model for *Tp53* and Wnt signaling resulted in MB that recapitulated the human MB subtype [4]. Recently, a mouse model for the most aggressive subgroup of human MB could be developed by enforced expression of *Myc* in *Tp53*-deficient cerebellar neuronal progenitors [5].

Retinoblastoma protein (Rb) exhibits tumor suppressive activity by binding and repressing a cell cycle activator, E2F (reviewed in [6]). Mutations of the *Rb* gene are rarely found in samples of human MB patients [7], and *Rb* knockout mice are predisposed primarily to the development of pituitary cancer rather than MB [8, 9]. However, simultaneous loss of function of *Rb* and *Tp53*, specifically in the EGL, had primarily shown typical MB development, strongly arguing that these genes and/or the signaling pathways regulated by these genes play critical roles in MB development [7]. The *CDKN2A* locus produces alternatively-spliced genes including *p14^ARF^* and *p16^Ink4a^* which up-regulate *TP53* function by binding MDM2 or inhibit cyclin-dependent kinase 4 and 6 (CDK4 and CDK6) through the attenuation of Rb phosphorylation, respectively. The loss of function deletions of *p14^ARF^* and *p16^Ink4a^* have been well characterized in diverse types of tumors (reviewed in [10, 11]). The gene *p15^Ink4b^*, on the other hand, though it is encoded by a related locus *CDKN2B* and displays high similarity to p16^Ink4a^ at the amino sequence level (˜85%), remains unclear in various tumorigenic processes.

During the last decade or so, several zebrafish cancer models were successfully generated, which recapitulated human cancers such as leukemia, neuroblastoma, and melanoma [12]. In an unprecedented way, the identification of novel cancer signaling pathways and visualization of pathological processes has been made possible by advances in optical clarity of zebrafish for high resolution imaging, chemical screening using whole animals, and genetic manipulations to generate mutants using genome editing tools such as Transcription activator-like effector nucleases (TALEN) and CRISPR/Cas9, as well as transgenic animals using Tol2 system and I-SceI meganuclease [13–15]. Despite these advancements, a zebrafish model for the most frequently occurring pediatric tumor, MB, is currently unavailable. Very recently, zebrafish was employed in somatic inactivation of *rb1* using TALEN to evaluate gene candidacy as a tumor suppressor [16]; however, the tumor types generated were not identified in detail, thus evaluating the interaction of two or more candidate tumor suppressor genes using somatic inactivation has not been reported yet, thus far.

In this study, we developed zebrafish cancer models by TALEN-mediated somatic inactivation of tumor suppressor genes with high efficiency. While *cdkn2a/b* gene inactivation accelerated development of Malignant Peripheral Nerve Sheath Tumors (MPNSTs) by TALEN injection in *tp53 ^e7/e7^* mutation background, TALEN-mediated somatic inactivation of *rb1*, which is a potential downstream target of *CDKN2A/B*, induced distinct brain tumors in the *tp53 ^e7/e7^* mutation background. Using RNA sequencing analysis together with histopathology and immunohistochemistry, we have demonstrated that brain tumors induced by *rb1* somatic inactivation have a molecular feature of MB-like primitive neuroectodermal tumors (PNETs).

## RESULTS

### Somatic inactivation of *cdkn2a/b* gene by the injection of TALEN mRNA leads to MPNSTs in F0 founder *tp53 ^e7/e7^* mutant zebrafish

**Figure 1:**
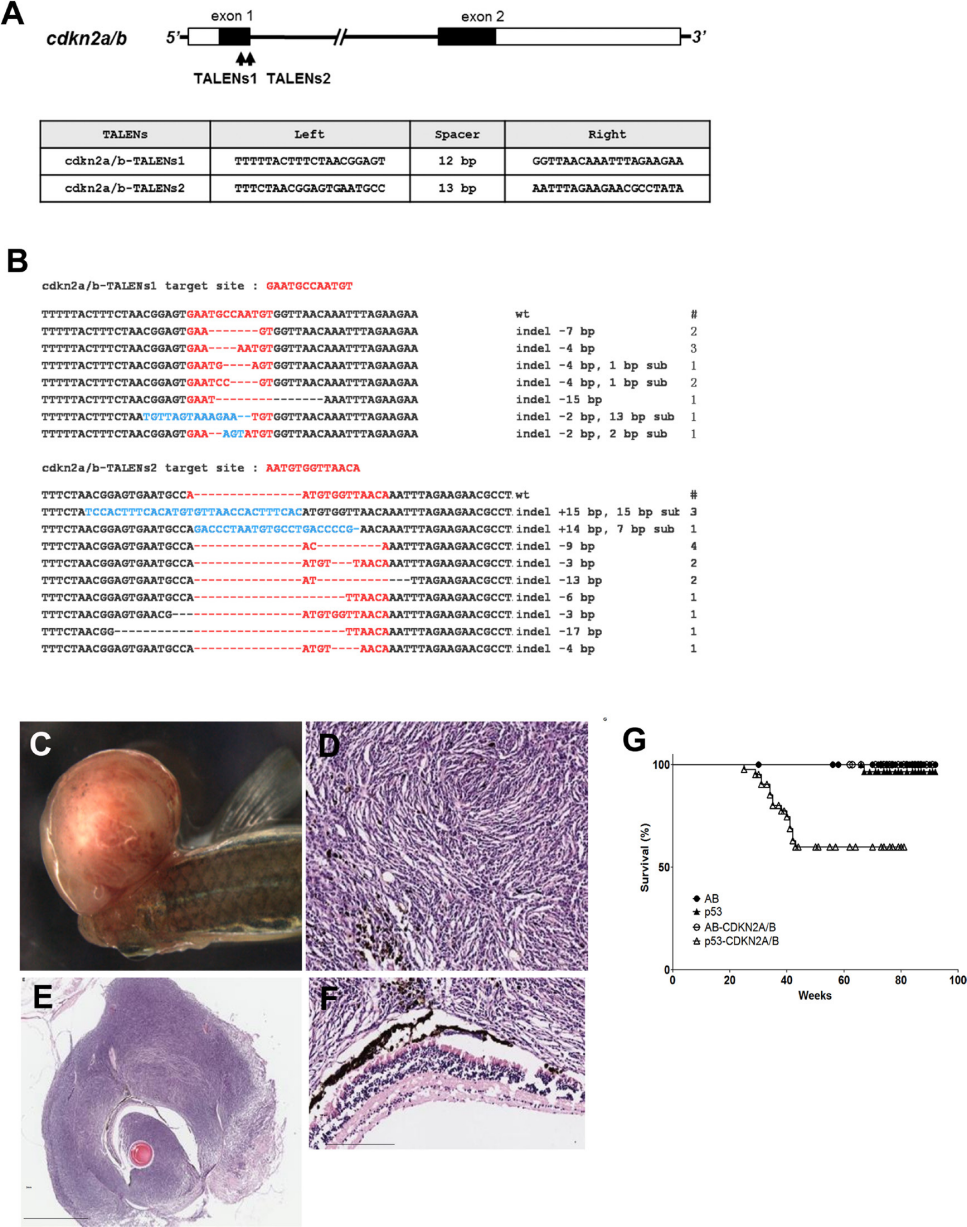
Somatic inactivation of *cdkn2a/b* gene by the injection of TALENs mRNA leads to MPNSTs in F0 founder zebrafish (**A**) The targeted regions of two different TALENs (*cdkn2a/b* TALENs-1 and *cdkn2a/b* TALENs-2) are located in exon 1 of *cdkn2a/b* gene. The binding sequences of TALENs for *cdkn2a/b* gene are also shown. (**B**) By the injection of *cdkn2a/b* TALENs-1 mRNA, 7 different germline mutated alleles of *cdkn2a/b* gene were recovered at F1 generation. And 9 different alleles were also recovered at F1 generation after injection of *cdkn2a/b* TALENs-2 mRNA. (**C**) The large mass of tumor was observed in adjacent to eyes of the *cdkn2a/b* TALENs mRNA injected *tp53* mutant zebrafish at 5.5 month post fertilization. (**D**–**F**) Histological image of cross sections of tumor tissue was visualized with Hematoxylin-Eosin staining. (D) High magnification image of tumor tissue shows the herringbone pattern that is a typical feature of malignant peripheral nerve sheath tumor. (F) There is no evident abnormality in the neuroepithelial layer of retina adjacent to tumor tissue. (**G**) Kaplan-Meier survival representation of wild type (AB), *tp53* mutant, and *cdkn2a/b* TALENs mRNA injected AB or *tp53* mutant zebrafish. The *cdkn2a/b* TALENs mRNA injected *tp53* mutant zebrafish had only started to exhibit death with tumor bearing as early as about 5 months. Other adult zebrafish such as *tp53* mutant and *cdkn2a/b* TALENs mRNA injected AB zebrafish did not show early death with tumor bearing. All data was acquired from two independent experiments. Scale bars: 2 mm (E) and 100 μm (D and F).

Synteny analysis demonstrated that zebrafish *INK4a*/*ARF*/*INK4b* locus was partially disrupted despite conservation of the adjacent gene block order (Supplementary Figure 1). Hence, zebrafish CDKN2A/B is the only encoded protein comparable to the three unique p19^ARF^, p16^INK4b^, and p15^INK4b^ proteins which are expressed from *INK4a*/*ARF*/*INK4b* locus in humans. To investigate the role of *cdkn2a/b* in tumorigenesis of zebrafish, we performed genetic inactivation by TALEN-mediated genome editing. Two different TALENs targeting the first exon of zebrafish *cdkn2a/b* gene (designated as *cdkn2a/b*-TALENs1 and *cdkn2a/b*-TALENs2, respectively) were constructed and followed by microinjection of *in vitro* synthesized TALEN mRNAs into one-cell stage zebrafish embryos (Figure 1A). After 3–4 months, founder zebrafish were successfully obtained which transmitted germline mutations to F1 progeny. With the injection of *cdkn2a/b*-TALENs1 mRNA, eleven independent F1 heterozygous zebrafish were recovered in which seven different alleles (6 frameshifts and 1 in-frame) were mutated in the *cdkn2a/b* gene of zebrafish. After injection of *cdkn2a/b*-TALENs2 mRNA, sixteen independent F1 heterozygous zebrafish were recovered in which nine different alleles (4 frameshifts and 5 in-frames) were mutated in *cdkn2a/b* (Figure 1B). Homozygous *cdkn2a/b* mutant embryos that were generated by incrossing F1 heterozygous zebrafish (4bp deletion allele induced by *cdkn2a/b*-TALENs2) could not express *cdkn2a/b* transcript, which was quantified by real time RT-PCR with 5 days old embryos (Supplementary Figure 2).

During analysis of *cdkn2a/b* mutants, we encountered an unexpected observation that adult *tp53* mutant zebrafish via one-cell stage injection of *cdkn2a/b*-TALEN mRNA could develop tumor mass, mainly occurring adjacent to the eyes as early as 6 to 7 months of age (Figure 1C, Table 1). Previous studies have reported that malignant peripheral nerve sheath tumors (MPNSTs) spontaneously developed in *tp53^e7/e7^* mutant zebrafish at 28% of tumor incidence rate by around 14 months of age [17, 18]. Similarly, our results showed that *tp53* mutant zebrafish could develop MPNSTs at an average of 66 weeks post fertilization (wpf) with a tumor incidence frequency of about 15 % (Table 1). Under the same conditions, injection of *cdkn2a/b*-TALEN mRNA led to an increase in tumor incidence frequency up to about 39% and accelerated tumor onset as early as 35 to 37 wpf in *tp53^e7/e7^* mutant zebrafish (Table 1). Kaplan–Meier survival curves also represented that *cdkn2a/b*-TALEN mRNA injection lead *tp53^e7/e7^* mutant zebrafish to early death with tumor bearing compared to non injected mutant zebrafih, remarkably (Figure 1G, *P* < 0.0001).

**Table 1:** The frequency and occurrence regions of tumors induced by somatic gene inactivation with *cdkn2a/b* or *rb1* TALENs in adult zebrafish

TALENs and genetic background	Average age of appearance of tumors (weeks)	Tumor frequency (%)	Tumor occurrence regions and types			
			Ocular MPNSTs	Abdominal MPNSTs	MB-like PNETs	Mixtures of MPNSTs & MB-like PNETs
WT ^a^	-	0/39 (0)	-	-	-	-
*tp53* mutant ^b^	66	5/34 (14.7)	1	1	-	-
WT-*cdkn2a/b* TALENs1	-	0/26 (0)	-	-	-	-
*tp53* mutant -*cdkn2a/b* TALENs1^c^	35	9/23 (39.1)	6	3	-	-
WT-*cdkn2a/b* TALENs2	-	0/29 (0)	-	-	-	-
*tp53* mutant-*cdkn2a/b* TALENs2^d^	37	7/19 (36.8)	4	3	-	-
WT-*rb1* TALENs1	32	7/30 (23.3)	-	-	7	-
*tp53* mutant-*rb1* TALENs1^e^	29	19/33 (57.5)	1	1	15^*1^	2
*tp53* mutant-*cdkn2a/b* TALENs2 &-*rb1* TALENs1^f^	31	20/42 (47.6)	4	1	8	7^*2^

Tumor frequency was measured at from 20 until 90 weeks post fertilization (wpf) except for *cdkn2a/b* TALENs2 and *rb1* TALENs1 double injection into *tp53* mutant, which was measured for 60 wpf^f^.^a^ wild type zebrafish is Oregon AB genetic background.^b^ Homozygous *tp53^e7/e7^*, tp53 M214K missense mutation in the p53 DNA binding domain preventing activation of p53.^c, d, e^ Adult homozygous *tp53^e7/e7^* zebrafish injected with about 200 pg of TALENs mRNAs for *cdkn2a/b* TALENs1, *cdkn2a/b* TALENs2, and *rb1* TALENs1, respectively. Data were obtained by two independent injection experiments.^*1^ Actually, 8 of 17 adult zebrafish with tumors in brain region were confirmed with histopathological methods.^*2^ 4 of 7 brain tumor-bearing zebrafish had ocular MPNSTs, and 3 of 7 brain tumor-bearing zebrafish had abdominal MPNSTs. Abbreviations: MPNST (Malignant peripheral nerve sheath tumors), MB (Medulloblstoma), and PNET (Primitive neuroectodermal tumors).

### Histopathological and immunohistochemical analysis of MPNSTs induced by somatic inactivation of *cdkn2a/b* gene in *tp53^e7/e7^* mutant zebrafish

Histopathological analysis by H & E staining of tumor tissues from *cdkn2a/b*-TALEN mRNA injected *tp53^e7/e7^* mutant zebrafish showed that spindle-shaped hyperchromatic nuclei were densely arranged in wave-like manner (Figure 1D, 1E). There was no evident abnormality in the neuroepithelial layer of retina adjacent to the tumor tissue (Figure 1F); also herringbone pattern of tumor tissue could be observed in Figure 1E as a typical feature characteristic of MPNSTs. We further analyzed tumor tissues from *cdkn2a/b*-TALEN mRNA injected *tp53^e7/e7^* mutant zebrafish with immunostaining methods.

**Figure 2:**
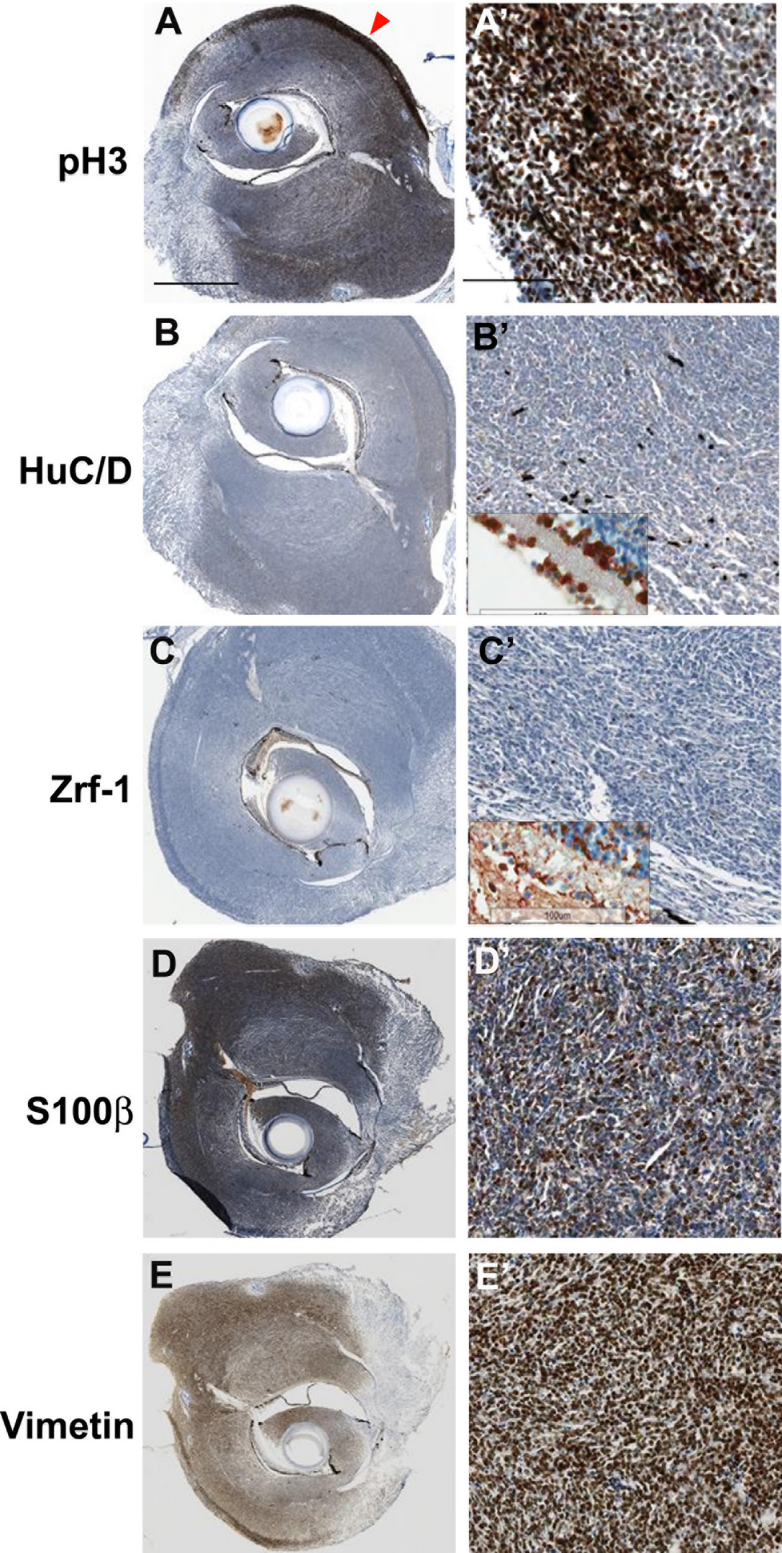
Immunohistochemical analysis of tumors from *cdkn2a/b* TALENs mRNA injected *tp53* mutant zebrafish (**A**) (**A’**) Immunostaining with anti- phospho-Histone 3 antibidy. The tumor tissue from *cdkn2a/b* TALENs mRNA injected *tp53* mutant zebrafish is highly mitotic. The phospho-Histone 3 positive mitotic cells were more evident in the outer region of tumor tissue (red arrowhead in A). (**B**) (**B’**) Neuronal signature that was visualized by immunostaining with anti-HuC/D antibody was scarcely detected in densely cellular region of tumor tissue. (**C**) (**C’**) Immunostaining with zrf-1 which detect zebrafish GFAP protein. The tumor tissue induced with *cdkn2a/b* TALENs mRNA also did not show any glial characteristics. Normal staining of anti-HuC/D and zrf-1 antibody could be observed in retinal layers near to tumor tissues (Inset box in B’ and C’). (**D**) (**D’**) Epithelioid MPNST marker, S100β, is strongly expressed in tumor tissue. (**E**) (**E’**) Mesenchymal marker, Vimentin, is also positively stained in tumor tissue. Scale bars: 1 mm (A) and 100 μm (A’).

Immunostaining with anti-phospho-Histone3(H3) antibodies demonstrated that tumor tissue of *cdkn2a/b* TALEN mRNA injected *tp53^e7/e7^* mutant zebrafish had highly mitotic cells (Figure 2A, 2A’). As phospho-H3 positive mitotic cells were more evident in the outer regions, it appeared that tumor tissues had outgrown radially from the tumor. To discern neural and/or glial cell origin, tumor tissues were stained with anti-HuC/D and Zrf-1 antibodies. Despite clear staining in normal retinal layers near the tumoral tissue (inset box in Figure 2B’ and Figure 2C’), anti-HuC/D positive neuronal signatures and Zrf-1 positive glial characteristics were scarcely detected in the dense cellular region of tissue (Figure 2B, 2B’, 2C, and 2C’). Despite limited diagnostic utility and variable staining (of about 50–90% of tumors) [19], S100 has traditionally been regarded as the best marker for human MPNST. Immunostaining with epithelioid MPNST marker, S100β, showed strong expression in spindle-shaped tumor cells (Figure 2D, 2D’) while the mesenchymal marker, Vimentin, also showed extensive staining in tumor tissues of *cdkn2a/b* TALEN mRNA injected *tp53^e7/e7^* mutant zebrafish (Figure 2E, 2E’). These data shows that TALEN-mediated somatic gene inactivation would be a useful strategy for tumor induction in zebrafish; targeted somatic inactivation of *cdkn2a/b* genes with TALENs efficiently induced early onset of MPNSTs with higher rate of incidence in *tp53^e7/e7^* mutant zebrafish.

### Somatic inactivation of *rb1* by TALEN mRNA injection induced tumors in head regions of F0 founder zebrafish

As was introduced above, zebrafish *cdkn2a/b* gene is a unique orthologue for the human *INK4a*/*ARF*/*INK4b* locus. According to reports, zebrafish CDKN2A/B may regulate CDK4/6-mediated phosphorylation of retinoblastoma (Rb) family proteins given that p15^INK4b^ and p16^INK4a^ maintain Rb family proteins in a hypophosphorylated state to promote E2F binding and G1 cell cycle arrest [11]. Thus, it seemed worthwhile to examine how *rb1* function in tumorigenesis compared to that of *cdkn2a/b* in zebrafish.

**Figure 3:**
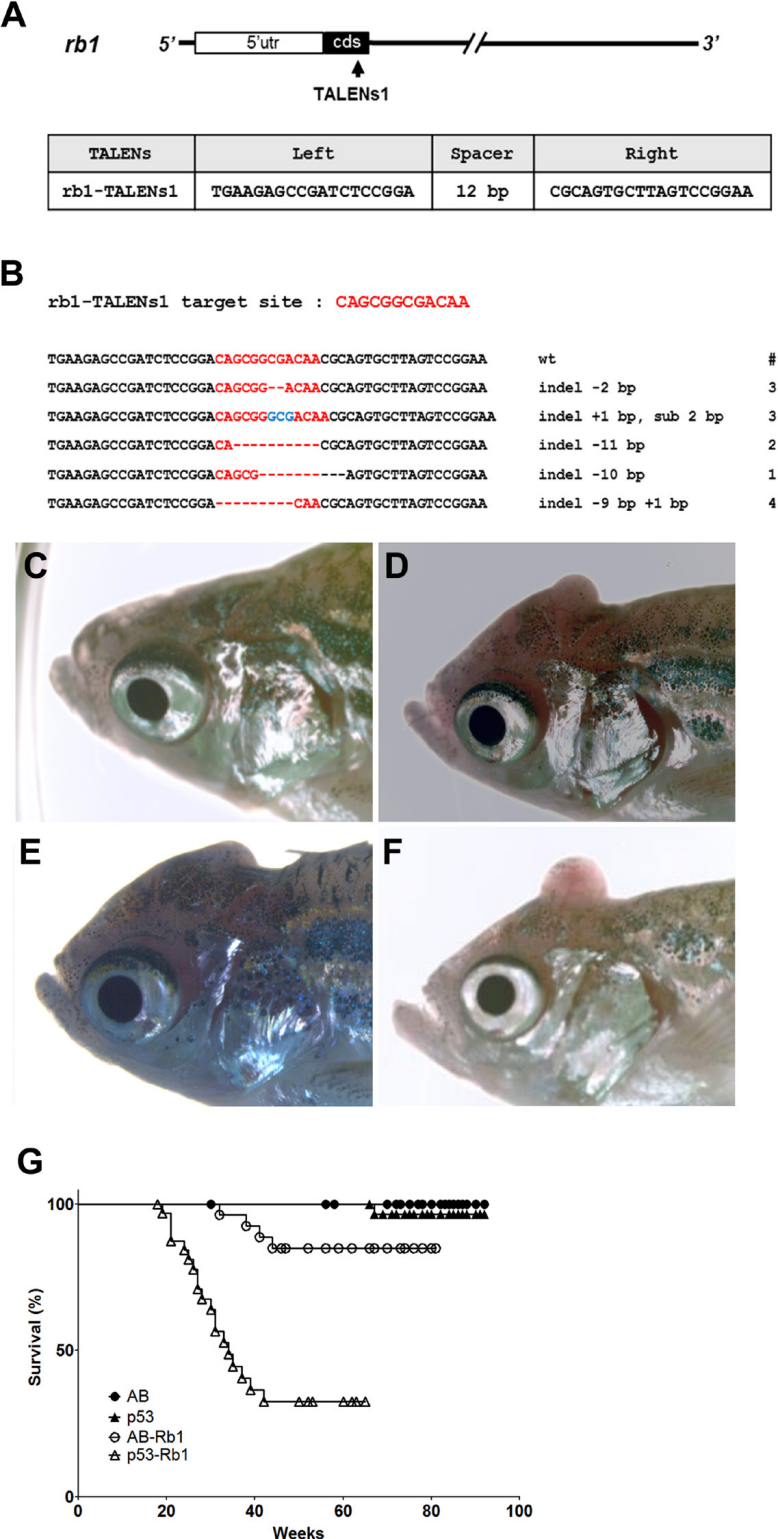
Somatic inactivation of *rb1* by the injection of *rb1*-TALENs mRNA leads to medulloblastoma like PNETs in F0 founder zebrafish (**A**) The targeted region of *rb1*-TALENs is located in 1st exon of zebrafish *rb1* locus. The binding sequences of TALENs for *rb1* are indicated. (**B**) After the injection of *rb1*-TALENs mRNA, 4 different deletion and 1 insertion germline mutated alleles of *rb1* were successfully recovered at F1 generation. Hence, 13 independent lines which have frameshift mutation in *rb1* could be obtained. (**C**–**F**) Morphological appearance of tumor bearing in head region of 5 month old zebrafish was presented. Protruding tumor mass were observed in *rb1*-TALENs mRNA injected zebrafish. The same aged wild type (C) and mutant (D–F) zebrafish are shown for comparison. (**G**) Kaplan-Meier survival representation of wild type, *tp53* mutant, and *rb1*-TALENs injected wild type or *tp53* mutant zebrafish. At about 6 month post fertilization, zebrafish which were injected with *rb1*-TALENs mRNA at one cell stage had started to die with tumors in the head region in wild type adult zebrafish. The tumor incidence which was induced by the injection of *rb1*-TALENs mRNA was more evident in *tp53* mutant zebrafish. Adult zebrafish that showed abnormal swimming behavior started to die with tumor in head regions as early as about 20 weeks after injection of *rb1*-TALENs mRNA into one cell stage embryos.

To examine the role of *rb1* in tumorigenesis of zebrafish, TALENs were designed to target the first exon of zebrafish *rb1* (designated as *rb1*-TALENs1). Somatic gene inactivation was performed using microinjection of *rb1*-TALENs1 mRNA into one-cell stage zebrafish embryos (Figure 3A). After injection, 13 independent germline-transmitted heterozygous zebrafish lines with frameshift mutations could be obtained in the F1 generation. Among them, 4 different deletion and 1 insertion mutated alleles of *rb1* were successfully recovered (Figure 3B). Homozygous *rb1* mutant larvae were generated by incrossing heterozygous F1 zebrafish with same frameshift mutated alleles. Because the expression of *rb1* gene was scarcely detected compared to wild type embryos at 5 dpf, homozygous *rb1* mutant had no functional Rb1 protein (Supplementary Figure 2). Homozygous mutants exhibited flattened swimming bladder, and enlarged liver at 7 day post fertilization (dpf) and did not survive until 10 dpf (Supplementary Figure 3).

Similar to *cdkn2a/b* inactivation of F0 *tp53^e7/e7^* mutants, TALEN-mediated somatic inactivation of *rb1* led to early onset of brain tumors in adult zebrafish. At around 4 to 5 months post fertilization, adult zebrafish injected at one-cell stage often displayed abnormal behaviors such as whirling or screwed swimming (data not shown). Additionally, tumor masses were visible starting from the posterior head region and protruding across the skull (Figure 3D–3F). The average age of tumor incidence was 7.5 months after birth, and 7 of 30 (23.3%) wild type zebrafish injected with *rb1*-TALEN mRNA at one-cell stage died bearing tumors in head regions (Table 1). Tumor induction was significantly accelerated by *rb1*-TALENs in the *tp53^e7/e7^* mutant background. 19 of 33 (57.5 %) of *rb1*-TALEN mRNA injected *tp53^e7/e7^* mutant zebrafish displayed tumors mainly in head regions while the average age of tumor incidence advanced about 1 month sooner than wild type background (Table 1). The Kaplan–Meier survival plot also showed decreased survival rate of *tp53^e7/e7^* mutants compared to wild type zebrafish injected with *rb1* TALEN mRNA at one cell stage (Figure 3G, *P* < 0.0001).

Targeted inactivation of *rb1* resulted in the early onset of brain tumors in zebrafish with significantly increased frequency of tumor incidence. These data also demonstrated the effectiveness of tumor induction with *rb1*-TALEN mediated somatic gene inactivation.

### Zebrafish tumors induced by somatic inactivation of *rb1* displayed characteristics of medulloblastoma-like PNETs

**Figure 4:**
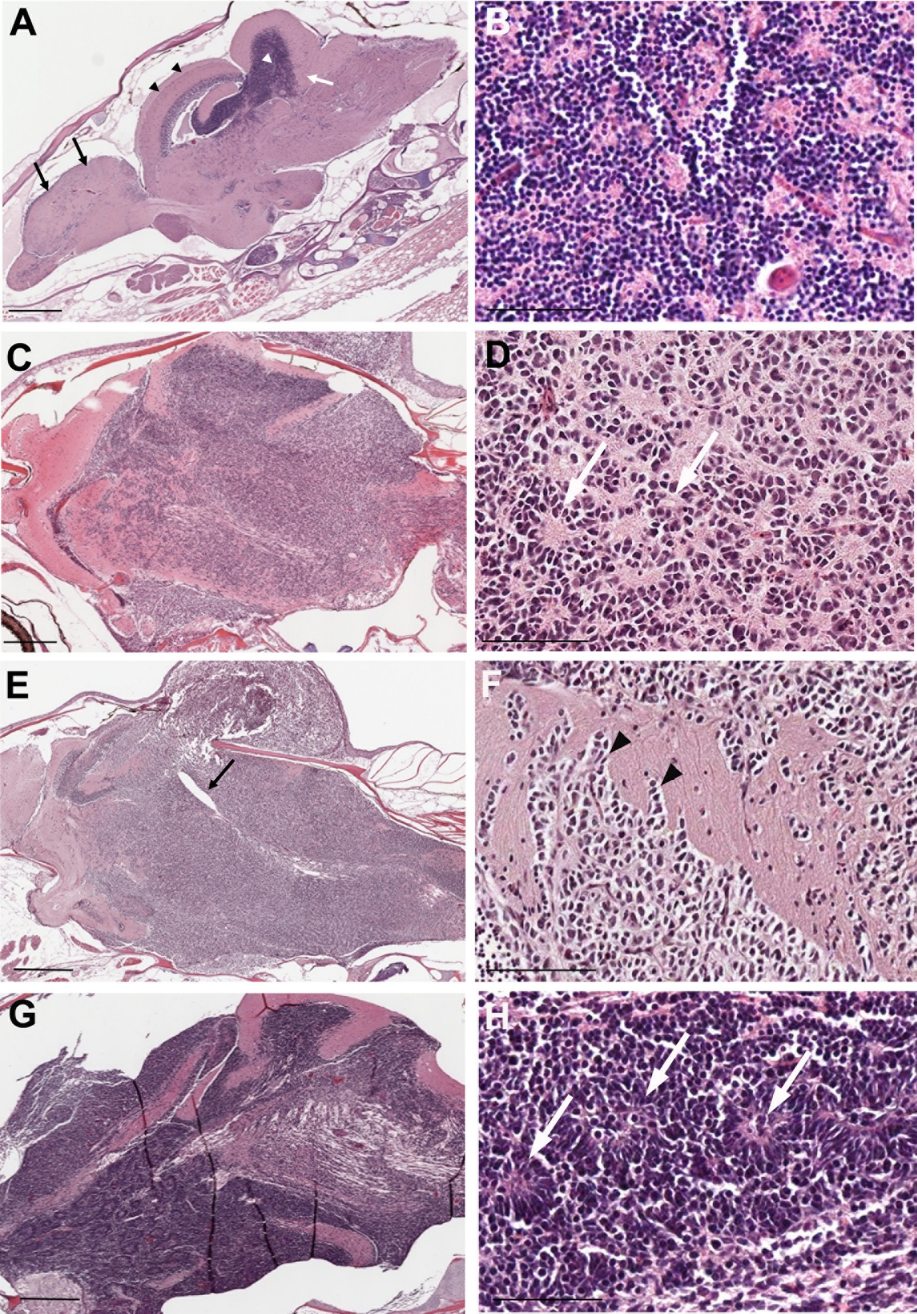
Histopathology of tumors from *rb1*-TALENs injected *tp53* mutant zebrafish (**A**) Sagittal section image of H & E staining of wild type zebrafish at 5 month post fertilization. Black arrows and arrow heads indicate forebrain and optic tectum, respectively. Cerebellum and cerebellar granule cells are indicated by white arrows and arrow heads, respectively. (**B**) High magnitude image of A. Normal cerebellar granule cells have a unique feature that is characterized with small rounded nuclei. (**C**–**H**) Sagittal section images of H & E staining of tumors from *rb1*-TALENs injected *tp53* mutant zebrafish. (C) Tumors were mainly arising in cerebellum, medulla, and brainstem. (D) High magnitude image of C. Highly cellular tumor cells with wedged nuclei were observed. Rosette like structures was marked by white arrows. (E) Tumors were mainly arising in cerebellum encompassed a majority of medulla, hypothalamus, and brainstem. The forth ventricle which were surrounded with tumor cells were marked by black arrows. (F) High magnitude image of E. Infiltration of tumor cells was indicated by black arrowheads in dorsal brainstem. (H) High magnitude image of G. Homer-Wright rosettes which are seen in PNETs or medulloblastomas were observed distinctly (white arrows). Anterior is left of all images. Scale bars: 500 μm (A, C, E and G), and 50 μm (B, D, F and H).

H & E staining was performed to analyze tumor tissues from *rb1*-TALEN mRNA injected *tp53^e7/e7^* mutant zebrafish. As reported previously [20] and shown in Figure 4A, the majority of H & E staining positive nuclei were located in the cerebellum of zebrafish brain. Normal cerebellar granule cells possess a unique feature characterized by small rounded nuclei. The stains showed nuclei gathered densely in the cerebellum granular layer (Figure 4B). Tumors from *rb1*-TALENs mRNA injected *tp53^e7/e7^* mutant zebrafish mainly arose in the cerebellum and encompassed a majority of the medulla, hypothalamus, and brainstem; resulting in abnormal brain structures (Figure 4C, 4E, 4G). Tumor cells visualized with H & E staining exhibited essentially similar appearance among the different samples. Tumor tissues were composed of highly dense tumor cells with hyperchromatic and wedged nuclei while definite Homer-Wright like structures were also detected in most dissected sections (white arrows in Figure 4D, 4F, 4H). In some sections, we observed that partial tumor cells could spread and infiltrate into adjacent normal tissue (black arrowheads in Figure 4F).

**Figure 5:**
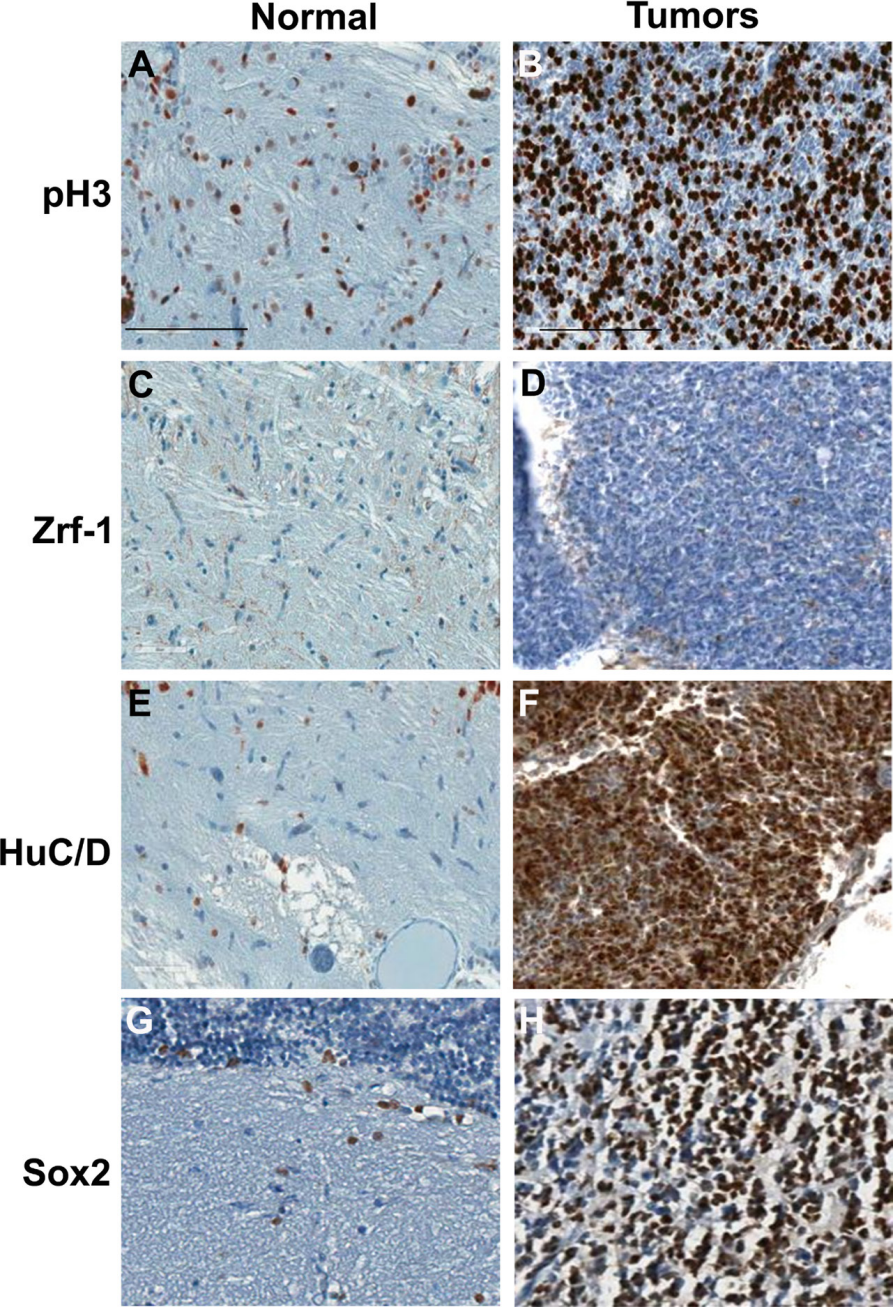
Immunohistochemical analysis of tumors from *rb1*-TALENs injected *tp53* mutant zebrafish Immunostaining was performed with lineage specific antibody with normal brain tissues (**A**, **C**, **E**, and **G**) and tumors from *rb1*-TALENs injected *tp53* mutant zebrafish (**B**, **D**, **F**, and **H**) using anti phospho-Histone 3 (A and B), anti Zrf-1 (C and D), anti HuC/D (E and F) and anti Sox2 (G and H) antibodies. Highly mitotic tumor cells could be detected in tumor tissues from *rb1*-TALENs mRNA injected *tp53* mutant zebrafish (B). Glial marker, Zrf-1 was scarcely stained in densely cellular region of tumor tissue (D). However, HuC/D antigen which is a postmitotic neuronal marker was strongly expressed in densely cellular region of tumor tissue (F). Neural progenitor marker, Sox2 expression was also detected in same cellular region of tumor tissue (H). Scale bars: 50 μm.

Two independent brain tumor tissues used as the serial sections in Figure 4C and 4E were further analyzed via immunohistochemistry for tumor lineage. About 50% of the tumor cells of *rb1*-TALENs injected *tp53^e7/e7^* mutant zebrafish displayed highly mitotic features (Figure 5B). We dismissed the possibility of glioma given that Zrf-1 positive glial characteristics were scarcely detected in the densely cellular region of *rb1*-TALEN induced brain tumor tissue (Figure 5D). Rather, the postmitotic neuronal marker, HuC/D antigen, was seen strongly expressed in this region of tumoral tissue (Figure 5F); and SOX2, a neural progenitor marker, was also detected. Taken together these data indicate that tumors from *rb1*-TALENs injected *tp53^e7/e7^* mutant zebrafish may be medulloblastoma-like PNETs based on their incidence location, histopathological features, and neuronal/neural characteristics from immunohistochemistry.

### *cdkn2a/b* or *rb1* were highly mutated in tumors induced from *cdkn2a/b-* or *rb1* TALEN-mRNA injection

**Figure 6:**
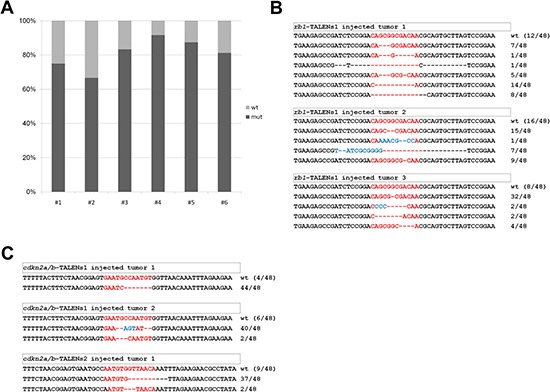
Mutational frequency and allele variation of *rb1* or *cdkn2a/b* genes in tumor tissues derived by TALENs injected F0 adult zebrafish (**A**) Mutational frequency of *rb1* or *cdkn2a/b* genes was evaluated from sequencing analysis of cloned amplicon from genomic DNA of independent tumor tissues induced by *rb1* or *cdkn2a/b* TALENs injected F0 *tp53* mutant zebrafish. Mutational frequency of tumor tissues from *rb1* TALENs injected F0 *tp53* mutant zebrafish were varied from 66.6% (32/48) to 83.3% (40/48), and that of tumor tissues from *cdkn2a/b* TALENs injected F0 *tp53* mutant zebrafish were varied from 81.3% (39/48) to 91.6% (44/48). (**B**) Mutation alleles of *rb1*'s exon 1 in tumor tissues from *rb1* TALENs injected F0 *tp53* mutant zebrafish. (**C**) Mutation alleles of *cdkn2a/b*'s exon 1 in tumor tissues from *cdkn2a/b*-TALENs1 or 2 injected F0 *tp53* mutant zebrafish.

To evaluate mutation frequency of *cdkn2a/b* or *rb1* in tumor tissues derived by TALEN injection, PCR products harboring each TALEN-targeted locus of genomic DNA were cloned and sequenced. The mutation frequency of *cdkn2a/b* or *rb1* from the tissues was higher than that of heterozygous mutants in all tested tumors. Mutation frequency in tumor tissues from *rb1* TALEN-injected F0 *tp53^e7/e7^* zebrafish varied from 66.6% (32/48) to 83.3% (40/48), while the frequency in *cdkn2a/b* TALEN-injected F0 *tp53^e7/e7^* zebrafish varied from 81.3% (39/48) to 91.6% (44/48) (Figure 6A).

Mutation forms of *rb1* in tumor tissues of *rb1* TALEN-injected F0 *tp53^e7/e7^* mutant zebrafish were analyzed. Total 16 mutations were identified from three independent tumor-bearing adult zebrafish. In two of the three tumors (*rb1*-TALEN-T2, *rb1*-TALEN-T3), all alleles showed frame-shift mutations; for the other tumor (*rb1*-TALEN-T1), 25% (9/36) of mutant alleles were in-frame mutations (Figure 6B). In the case of *cdkn2a/b*, most mutations in tumor tissues of *cdkn2a/b* TALEN-injected F0 *tp53^e7/e7^* zebrafish were also frame-shifts, while some were in-frame mutations (Figure 6C).

Furthermore, *cdkn2a/b* and *rb1* transcripts were also rarely expressed in tumors induced by *cdkn2a/b* and *rb1* somatic inactivation by TALENs injection, respectively (Supplementary Figure 2).

These data show that TALEN-mediated tumors were induced by loss-of-function of targeted-genes and that these loss-of-function mutations were resulted from the highly efficient bi-allelic inactivation of target genes.

### Molecular signatures of zebrafish tumors induced by *rb1* somatic inactivation displayed a similar characteristic of human MB and/or PNETs

QuantSeq 3′ mRNA sequencing was performed in order to characterize the molecular features of tumor tissues induced by *rb1*-TALEN injection. For QuantSeq 3′ mRNA sequencing, normalization (i.e. RPKM or TPM) for sequencing depth and gene length was not required since only one fragment per transcript is generated and also the number of reads mapped to a gene is directly linked to its expression [21]. To identify differentially expressed genes, we selected genes for which fold change was higher than 1.50 and lower than 0.67. We finally obtained 1679 up- and 1183 down-regulated transcripts for the tumor tissues (Supplementary Table 1).

**Figure 7:**
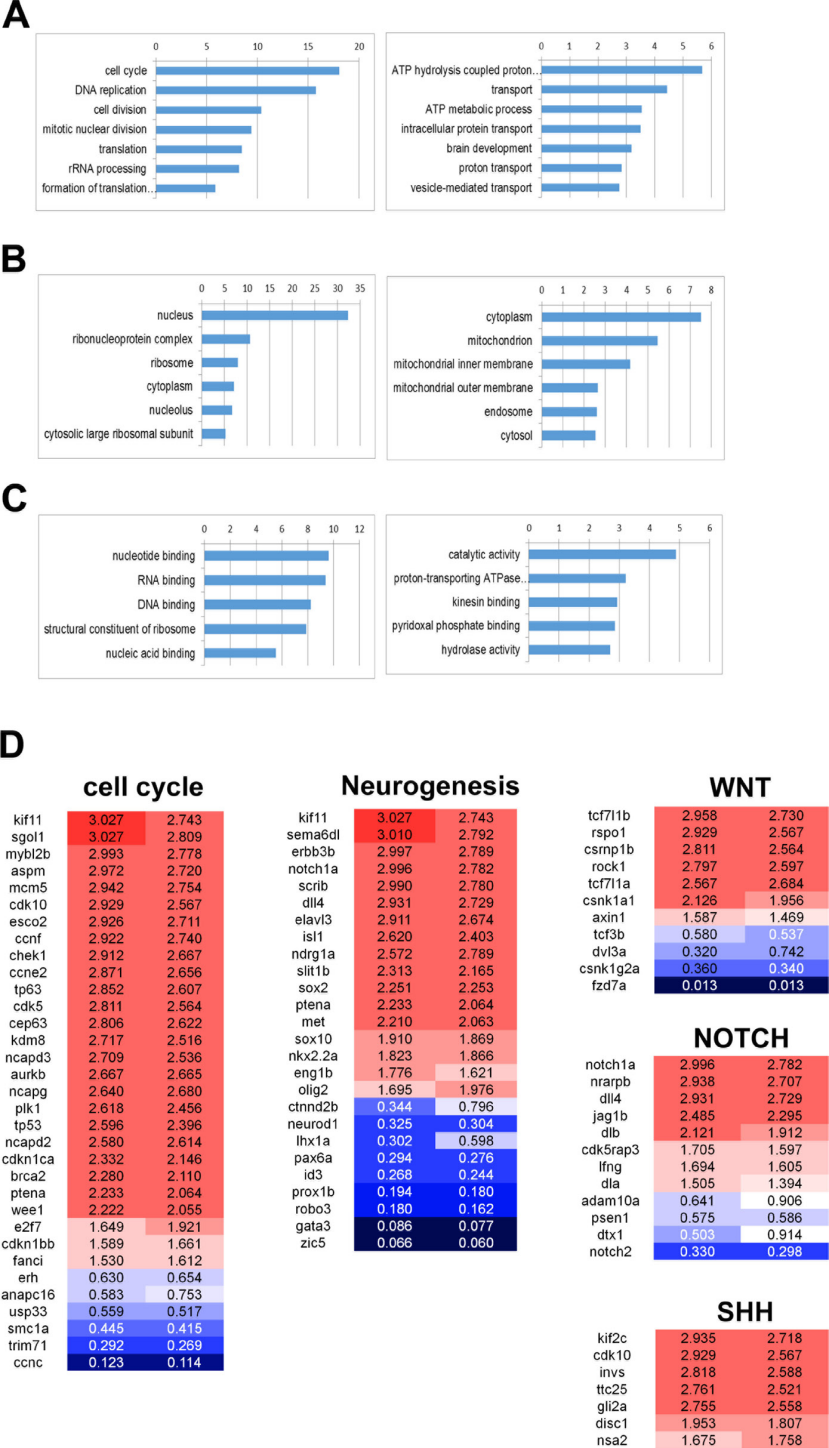
Gene ontology analysis of the differentially expressed genes in tumors induced by *rb1* somatic inactivation Gene Ontology Analysis was performed with the differentially expressed genes (DEG), after QuantSeq 3′ mRNA sequencing of tumors induced by *rb1* somatic inactivation. (**A**–**C**) -log10 (*P* value) was indicated at the top. (A) The up or down-regulated in biological processes. The cell cycle, DNA replication, cell division, and translation process were significantly up-regulated however ATP metabolism and vesicle/protein transport were down-regulated. (B) The up or down-regulated in cellular component. The nucleus and ribonucleoprotein complex were up-regulated. (C) The up or down-regulated in molecular function. (**D**) The featured DEGs in the neurogenesis, WNT, SHH, and NOTCH pathway. Red and blue represent up- and down-regulation of genes with fold change.

Gene Ontology Analysis was performed in order to understand the signatures of differentially expressed genes (DEGs). The analysis showed that cell cycle, DNA replication, cell division, and translation were significantly up-regulated while ATP metabolism and vesicle/protein transport were down-regulated in the tumor tissues. DNA binding in the nucleus, RNA binding, structural constituent of ribosome of molecular function and cellular component of gene ontology were also up-regulated in tumor tissues (Figure 7A–7C, Supplementary Table 2). Tumors induced by *rb1* somatic inactivation displayed medulloblastoma like PNET characteristics in histopathology and immunohistochemistry analysis (Figure 4, Figure 5). Thus, we further analyzed pathway signatures of DEGs and focused especially on neurogenesis, WNT, SHH, and NOTCH pathways which are used as subgroup classifications of human medulloblastoma [2]. A variety of genes involved in cell cycle progression were highly expressed in the tumors (Figure 7D). Interestingly, expression levels of differentiated neuronal genes (i.e. *elav3*, *isl1*, etc.) and undifferentiated neuronal precursor genes (i.e. *notch1a*, *sox2*, *nkx2.2a*, etc.) were coincidentally up-regulated as well (Figure 7D). Although GESA for WNT, SHH, and NOTCH pathways scarcely showed statistical significance (data not shown), we could observe the up-regulation of some WNT component genes (such as *tcf7l1a* and *tcf7l1b*), SHH target genes (such as *klf2c*, *invs*, and *gli2a*), and NOTCH receptor or ligand genes (such as *notch1a*, *dll4*, *dla*, and *dlb*) and target genes (such as *nrarpb*) (Figure 7D). Thus, these data demonstrated that molecular signatures of tumors induced by *rb1* somatic inactivation were characterized by high activation of cell cycle progression and protein synthesis and the up-regulation of neuronal genes including WNT, SHH, and NOTCH pathway components or target genes.

**Figure 8:**
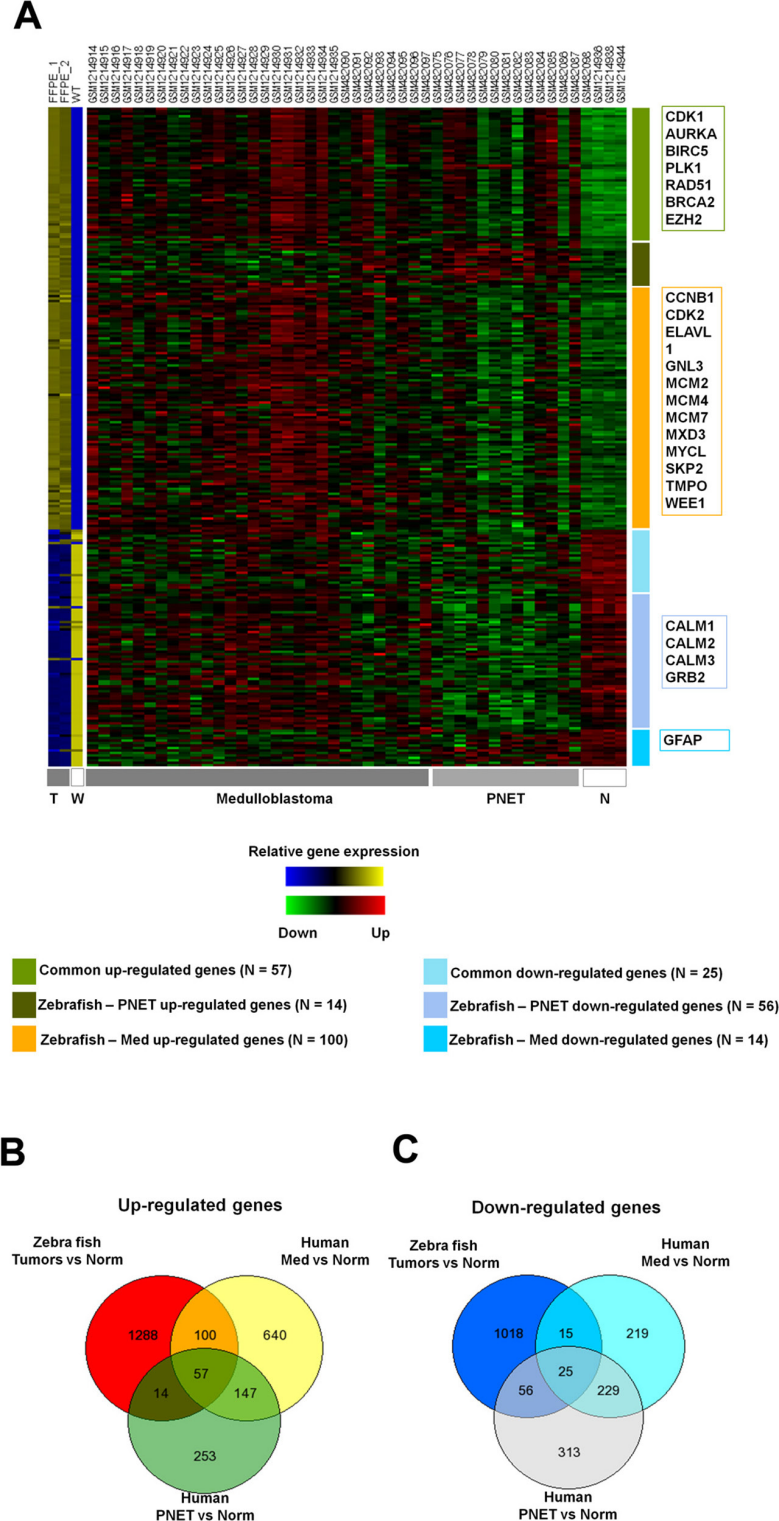
Comparative analysis between gene expression profiles of zebrafish tumors induced by rb1 somatic inactivation and public micro array data of human PNETs and medulloblastomas (**A**) Heatmap of gene expression profiles. (T = Tumors, W = Wild type, and N = Normal tissue) The individual samples are indicated at the top of each column and the group of samples is indicated in the bottom. The representative genes are indicated in boxes at the left. (**B**, **C**). Venn diagrams showing differently expressed genes. (B) up-regulated and (C) down-regulated genes. (FDR < 0.05) (Norm; Normal sample, Med; medulloblastoma, PNET; primitive neuroectodermal tumor).

Moreover, public microarray data of human medulloblastomas and PNETs were used to compare gene expression profiles of tumors induced by *rb1* somatic inactivation (Table 2). As presented in Figure 8A, there were 57 up-regulated genes common to the tumors induced by *rb1* somatic inactivation (zebrafish tumors) and human medulloblastomas and PNETs most of which were involved in cell cycle progression and mitosis (such as *cdk1*, *aurka*, *plk1*, etc., Supplementary Table 3). Of the up-regulated genes common to zebrafish tumors and human medulloblastomas, most of them are involved in cell cycle progression (*ccnb1*, *cdk2*, *mycl*, *wee1*, etc.) and/or DNA replication, initiation, and elongation (*mcm2*, *mcm4*, *mcm7*, etc., Figure 8A, Supplementary Table 3) and primarily annotated to medulloblastoma associated genes (DisGeNET, http://www.disgenet.org/web/DisGeNET/menu/home) [22]. Interestingly, there were more simultaneously up-regulated genes (157 genes) in zebrafish tumors and human medulloblastomas than there were (71 genes) in zebrafish tumors and human PNETs (Figure 8A, 8B). On the other hand, simultaneously down-regulated genes (40 genes) in zebrafish tumors and human medulloblastomas were fewer than those (81 genes) in zebrafish tumors and human PNETs (Figure 8A, 8C). These findings demonstrated that tumors induced by *rb1*-TALEN mediated somatic inactivation have both medulloblastoma and PNET molecular characteristics albeit a greater number of medulloblastoma associated up-regulated genes than PNET up-regulated genes.

**Table 2:** A list of the collected affymetrix human genome U133 Plus 2.0 array samples using the comparative analysis

GSE	Raw data	Normal sample	Tumor sample	PNET	Medulloblastoma
GSE19404	24	1	21	13	8
GSE50161	130	3	22	-	22

## DISCUSSION

Somatic gene inactivation of tumor suppressor genes is a general feature of most human tumors [23–26]. In animal tumor modeling, however, most general methods for tumor suppressor gene inactivation involved germline-targeted knockout strategies [27, 28]. In the course of developing tumor suppressor gene knockout progeny, we observed that somatic gene inactivation of *cdkn2a/b* and/or *rb1* efficiently induced tumors in the F0 founder generation. Our observations are consistent with previous reports that brain tumor modeling could be successfully performed with TALENs or CRISPR/Cas9 mediated somatic gene inactivation for tumor suppressor genes in mouse and zebrafish [16, 29]. The extremely high mutation frequency of *cdkn2a/b* or *rb1* in tumor tissues derived by TALENs injection also supports the possibility of tumor induction by specific inactivation of *cdkn2a/b* or *rb1* (Figure 6). Taken together, our findings showed that somatic inactivation of tumor suppressor genes by genome editing tools such as TALENs or CRISPR/Cas9 engineered nucleases could be a useful method for the development of animal tumor models.

Gene *rb1* has important roles in regulating G1 to S phase entry by repressing the activity of E2Fs transcription factors and CDKs. Inactivation of *rb1* is thought to promote either induction or progression events in a variety of cancers such as retinoblastoma, prostate cancer, small cell lung cancer, etc. [30–34]. In this study, brain tumors induced by somatic inactivation of *rb1* showed incidence mainly in the cerebellum. Histopathology data exhibited distinct Homer-Wright like features [35] while immunohistochemistry elicited positive staining for neuronal/neural but not glial cell. For these reasons, we assumed that brain tumors induced by somatic inactivation of *rb1* are medulloblastoma like PNETs, which molecular comparative analysis of RNA-seq results confirmed (Figure 8). Although *rb1* mutation has not been widely detected in traditional human medulloblastomas, some reports from clinical case studies and animal tumor models have suggested that *rb1* pathway participates in medulloblastoma development. In rare cases, pediatric patients with hereditary retinoblastoma harboring *rb1* mutation are at an increased risk for pineoblastoma, supratentorial PNET, and medulloblastoma [36, 37]. Conditional knockout of *rb1* in cerebellar external granular cells of *tp53* knockouts induced development of medulloblastoma [7]. These reports are consistent with our findings that somatic inactivation of *rb1* induced medulloblastoma like PNETs and that frequency of tumor induction was noticeably enhanced in *tp53^e7/e7^* mutant zebrafish (Figure 3). A recent report showed that in glial cells of transgenic mice, overexpression of E2F1, a transcription factor downstream of *rb1* pathway, also resulted in the induction of medulloblastomas and other PNETs [38]. Interestingly, conditional knockouts of upstream or downstream regulators of *tp53^e7/e7^* - *ATM*, *p19^ARF^* (a mouse homolog of human *p14^ARF^*) or *p21*- were not able to elicit MB in *Rb* knockout background, indicating a specific role of *Tp53* (such as inducing genomic instability, rather than cell cycle regulation or DNA damage response) in MB/PNET pathogenesis [39].

In mammals, *p15^Ink4b^* encoded by the *cdkn2a* locus neighboring *cdkn2b* locus, was presumed to be generated by gene duplication and believed to play a redundant role for *p16^Ink4a^*. In zebrafish, there seems to be only one *cdkn2* gene, collectively called *cdkn2a/b*. Although mammalian *p15^Ink4b^* has been implicated primarily in leukemia and lymphoma [11], its involvement in MPNSTs has been suggested due to its expression and genetic status [40]. Consistent with this notion, vascular smooth muscle cells, the potential tumor cell origin for MPNST, underwent apoptosis in *p15^Ink4b^* knockout mice in a *tp53*-dependent manner [41]. Therefore, the acceleration of MPNSTs upon somatic inactivation of *cdkn2a/b* in *tp53^e7/e7^* mutant background may possibly be due to a reduction in apoptosis of affected tumor cells of origin. The exact tumor types induced by *cdkn2a/b* mutations would be determined according to binding partner and/or biological context in normal and cancer cells. It was notable that *cdkn2a/b* and *rb1* double somatic inactivation did not influence the overall tumor incidence rate, and merely lead to form the mixture of MPNSTs & MB-like PNETs in an each single tumor developing zebrafish (Table 1). These data mean that somatic inactivation of *cdkn2a/b* or *rb1* did not show a synergic effect on tumor incidence rate or tumor types each other, and could be postulated that *cdkn2a/b* and *rb1* have a distinct role in tumorigenesis.

The molecular features of zebrafish tumors induced by *rb1* somatic inactivation were characterized by an up-regulation of genes for cell cycle progression, DNA replication, and translation, and a down-regulation of genes for ATP metabolism and vesicle/protein transport (Figure 7). These molecular characteristics are well-conserved features of a variety of tumors including medulloblastoma and PNETs. Consistent with the data, the signatures of tumor DEGs induced by *rb1* somatic inactivation were closely associated with those of human medulloblastomas and PNETs (Figure 8). Hence, we could conclude that tumors induced by *rb1* somatic inactivation are medulloblastoma-like PNETs. According to several reports [4, 42–45], medulloblastoma is classified into four molecularly distinct subgroups –WNT, SHH, Group C (MYC activated), and Group D. Zebrafish tumors induced by *rb1* somatic inactivation displayed an apparent up-regulation of genes in neural development and NOTCH pathway. Some genes of the WNT and SHH pathways were specifically up-regulated in zebrafish tumors but not to statistical significance. Therefore, we inferred that zebrafish tumors induced by *rb1* somatic inactivation are a primitive type of medulloblastoma-like PNET accompanied by indistinguishable subtypes.

In summary, we have presented the efficient development of a zebrafish tumor model by TALEN-mediated somatic inactivation of tumor suppressor genes. The development of MPNSTs was accelerated by *cdkn2a/b* gene inactivation via TALEN mRNA injection into fertilized eggs of *tp53^e7/e7^* mutant background. Also, TALEN-mediated *rb1* somatic inactivation led to the development of brain tumors in the zebrafish model. Using RNA sequencing analysis in addition to histopathological and immunohistochemistry, we showed that brain tumors induced by *rb1* somatic inactivation have molecular features of medulloblastoma-like PNETs.

## MATERIALS AND METHODS

### Wild type and mutant zebrafish

Wild type zebrafish (*Danio rerio*) with Oregon AB genetic background were used. The *tp53^e7/e7^* (*tp53^M214K^*) mutant was previously described [46]. Zebrafish were essentially maintained as described in [47], and all experimental procedures using zebrafish was approved by the Institutional Animal Care and Use Committee at National Cancer Center, Republic of Korea (permit number: NCC-15-116C).

### TALEN construction, mRNA injection, and mutational analysis

TALEN vectors targeting the first exon of *cdkn2a/b* gene (Figure 1A) and *rb1* (Figure 3A) were designed and constructed by ToolGen (http://toolgen.com/). TALEN vectors were linearized by PvuII, extracted with phenol/chloroform, and followed by ethanol precipitation. Subsequently, mRNAs encoding TALENs were synthesized using the mMESSAGE MACHINE T7 Transcription kit (Ambion) and purified as shown above; and about 200 pg of mRNAs were injected into one-cell stage zebrafish embryos.

Site-specific TALEN efficiency was examined by target-specific PCR with isolation of genomic DNA, and followed by T7 endonuclease I assay. In brief, the genomic DNA was extracted in DNA isolation buffer (10 mM Tris, 10 mM EDTA, 150 mM NaCl, 0.5% SDS and 0.1 mg/ml Proteinase K) for 2 h at 60°C, and PCRs were performed using the following primer pairs: *cdkn1a/b*-TALENs1&2 forward primer, 5′-CAGCGTTGAACTGATTGTTTTCG-3′; *cdkn1a/b*- TALENs1&2 reverse primer, 5′-TCCCATATAGTCAA ACAGGTGTG-3′; *rb1*-TALENs1 forward primer, 5′-CTG AGAGTGAACGCGCTCTTCT-3′; *rb1*-TALENs1 reverse primer, 5′-CGTCAGGTCGCTCTCTTCCTTCC-3′. Consequently, PCR products were purified by PCR/GEL purification mini kit (FAVORGEN) and analyzed by T7 endonuclease I assay or sequencing.

### Histopathology and immunohistochemistry

For observation of histological features of normal and tumor tissues, adult zebrafish were anesthetized with 0.016% of tricane methanesulfonate (Sigma), fixed in 4% paraformaldehyde in PBS for 16 h at 4°C, and then decalcification was followed as described previously [48]. After sectioning (4 to 6 mm) and pretreatment of paraffin embedded tissue, tissue sections were stained with hematoxylin (DaKo) and eosin (Merck). Laser capture microdissection with LMD6 (Leica) was used for normal or tumor tissue preparation to examine the mutation allele of genomic DNA or analyze the differential expression level of RNAs with RNA-seq.

For immunostaining, antigen retrieval process and endogenous peroxidase blocking was performed with citrate buffer (pH 6.0) and 3% hydrogen peroxide, respectively. Tissue sections were incubated in 5% normal serum in PBSBD (1% BSA and 1%DMSO in PBS) for 1 h at room temperature and were reacted with primary antibody overnight at 4°C in a humidified chamber. Anti-phospho-Histone H3 (1/100 dilution, mouse monoclonal, Sigma), anti-GFAP (also known as Zrf-1, 1/2000, mouse monoclonal, Zebrafish International Resource Center), anti- HuC/HuD (1/2000, 16A11, mouse monoclonal, Sigma), anti-S100β (1/100, rabbit monoclonal, Abcam), anti-Vimentin (1/500, rabbit monoclonal, Abcam), and anti-Sox2(1/1000, rabbit monoclonal, Abcam) antibodies were used. VECTASTAIN Elite ABC HRP Kit (Vector Laboratories) and 3, 3′-diaminobenzidine (Vector Laboratories) as a chromogen were used for visualization of signals.

### RNA isolation and library preparation and RNA sequencing

In order to characterize the molecular features of tumor tissues induced by *rb1*-TALENs injection, the tumor tissues were collected with laser captured microscope from paraffin embedded tissue section. Total RNA was isolated using Trizol reagent (Invitrogen). RNA quality was assessed by Agilent 2100 bioanalyzer using the RNA 6000 Nano Chip (Agilent Technologies), and RNA quantification was performed using ND-2000 Spectrophotometer (Thermo). Construction of the library was performed using SENSE 3′ mRNA-Seq Library Prep Kit (Lexogen) according to manufacturer instructions. In brief, reverse transcription reaction were performed with 500 ng total RNA and oligo-dT primer containing an Illumina-compatible sequence at its 5′ end. And then, synthesis of the second strand was initiated by random primer containing an Illumina-compatible linker sequence at its 5′ end, after degradation of the RNA template. The double-stranded library was purified by using magnetic beads to remove all reaction components, and amplified to add the complete adapter sequences required for cluster generation. High-throughput sequencing was performed as single-end 75 sequencing using NextSeq 500 (Illumina).

### Data analysis

About 9 to 10 million sequence tags (11.6×10^6^ for normal brain tissue, 9.8 and 8.9 × 10^6^ for two independent tumor tissues) were obtained. SENSE 3′ mRNA-Seq reads were aligned using Bowtie2 version 2.1.0 [49]. Bowtie2 indices were either generated from genome assembly sequence or the representative transcript sequences for aligning to the genome and transcriptome. After mapping to the zebrafish reference sequence database for quantitative analysis of transcripts expression, 8329 genes for normal brain tissue, 5403 and 5688 genes for two independent tumor tissues were annotated. Differentially expressed genes were determined based on counts from unique and multiple alignments using EdgeR within R version 3.2.2 (R development Core Team, 2011) in BIOCONDUCTOR version 3.0 [50]. The RT (Read Count) data were processed based on Quantile normalization method using the Genowiz ™ version 4.0.5.6 (Ocimum Biosolutions). Cytoscape (version 2.7, http://www.cytoscape.org/), an open source bioinformatics platform developed by the Institute of Systems Biology, was used to construct network diagrams and to illustrate clustering of the genes in our dataset within specific pathways. Gene classification was based on searches done by DAVID (http://david.abcc.ncifcrf.gov/) and Medline databases (http://www.ncbi.nlm.nih.gov/).

Microarray data was collected for medulloblastoma, primitive neuroectodermal tumor and normal samples from Gene Expression Omnibus [51]. The gene expression study sample dataset included 30 medulloblastomas, 13 primitive neuroectodermal tumors, four normal brain samples. Scanned microarray data were background corrected and normalized using the Robust Multi-Array Average algorithm resulting in log2 gene expression values. Differently expressed genes were selected using false discovery rate < 0.05 between tumor and normal samples. Human gene symbols were converted to zebrafish gene symbols using The Zebrafish Model Organism Database [52]. Python (version 2.7.6) and the Pandas python library (version 0.15.2) were used for most data analysis. R (version 3.1.0) and MeV (version 4.9.0) [53] were used for image production. BIOCONDUCTOR version 3.0 [54] package was used for normalization of microarray data.

## SUPPLEMENTARY MATERIALS FIGURES AND TABLES








