# Supplementary material for: Development of zebrafish medulloblastoma-like PNET model by TALEN-mediated somatic gene inactivation
Source: Oncotarget. 2017 Jul 21;8(33):55280–97. doi: 10.18632/oncotarget.19424 (PMC5589658; doi:10.18632/oncotarget.19424)
Supplement: Supplementary file 1 [file oncotarget-08-55280-s001.pdf]

Development of zebrafish medulloblastoma-like PNET model by TALEN-mediated somatic gene inactivation

SUPPLEMENTARY MATERIALS

MATERIALS AND METHODS

Real-time quantitative RT-PCR

Total RNA was isolated using TRIzol reagent (Thermo Fisher Scientific), and the cDNAs were synthesized by reverse transcriptase (RT) using iScript cDNA synthesis kit (Bio-Rad), according to the manufacturer's recommended protocol. Real-time quantitative PCR was performed using iQ SYBR Green supermix (Bio-Rad) with the following specific primers; *cdkn2a/b*, 5′- ACGTCGAGGATGAACTGACC -3′ (forward) and 5′- CTGTTCCAGCAGCACAAGAG -3′ (reverse); *rb1*, 5′- CCCCACAGAAGAAAGAGCTG -3′ (forward) and5′- TTGTCCATCGATCTGATCTCC -3′ (reverse); β-Actin, 5′- CGACAGGATGCAGAAGGAGA -3′ (forward) and 5′- TAGAAGCATTTGCGGTGGAC -3′ (reverse) on LightCycler 96 system (Roche). All real-time quantitative PCR experiments were performed in triplicate and quantification cycle (Cq) values were determined using the Graphpad Prism 7 (trial version) software. Relative quantification of the mRNA levels was performed using the Roche the E Method with β-Actin as a reference gene.

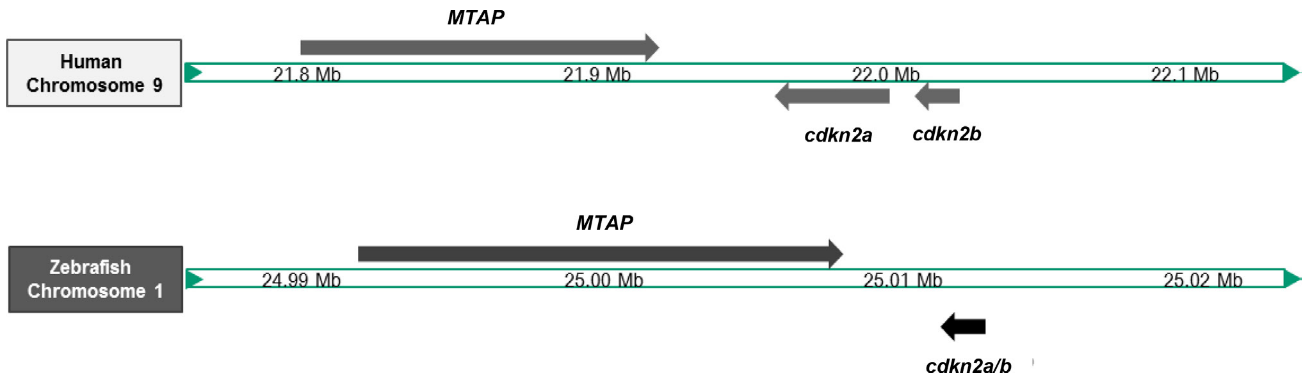

Supplementary Figure 1: Synteny analysis of *cdkn2a* and *cdkn2b* locus between zebrafish and human.

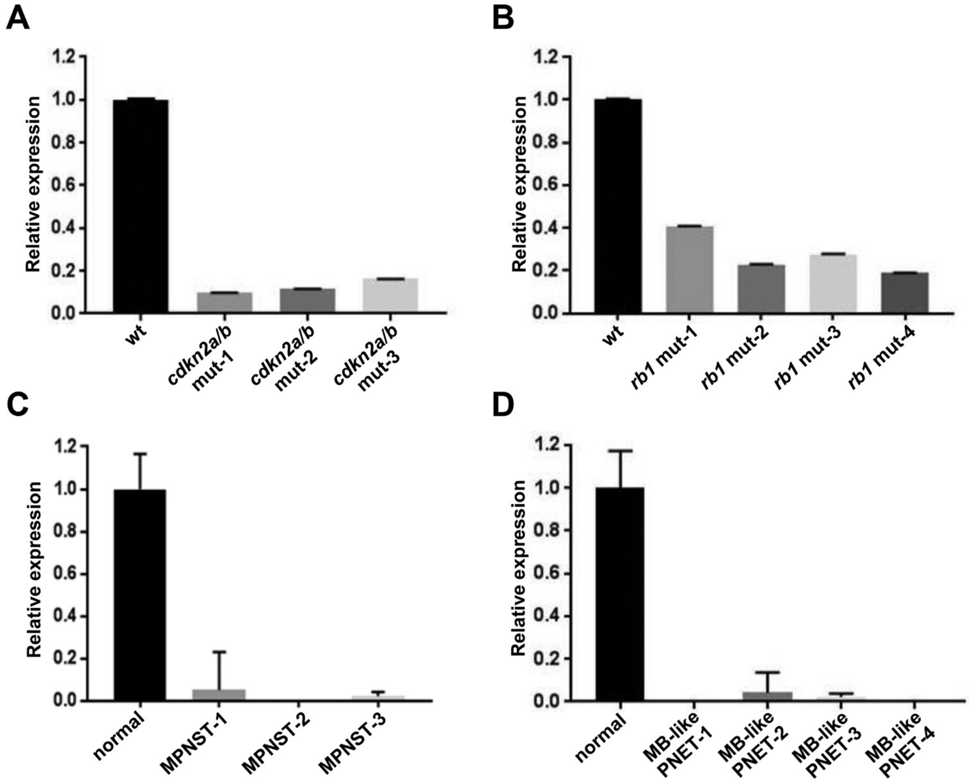

Supplementary Figure 2: The expression of *cdkn2a/b* and/or *rb1* were rarely detected in homozygous *cdkn2a/b* and/or *rb1* mutant embryos, and tumors induced by somatic inactivation of *cdkn2a/b* and/or *rb1* genes. (**A**–**B**) Quantitative RT-PCR analysis was performed with RNA from homozygous *cdkn2a/b* (A) and *rb1* (B) mutant (mut) embryos at 5 dpf, respectively. Homozygous *cdkn2a/b* and/or *rb1* mutant could not express their transcripts. (**C**–**D**) Quantitative RT-PCR analysis was performed with RNA from tumors induced by somatic inactivation of *cdkn2a/b* (MPNSTs) and/or *rb1* (MB-like PNETs). The expression of *cdkn2a/b* and *rb1* was rarely detcted in tumors, respectively. Relative expression of genes was calculated relative to the expression level of mRNA in wild type (wt) zebrafish embryos or adult head tissues (normal). All graphs represent two independent experiments performed in triplicate. Error bars represent standard deviations from the mean.

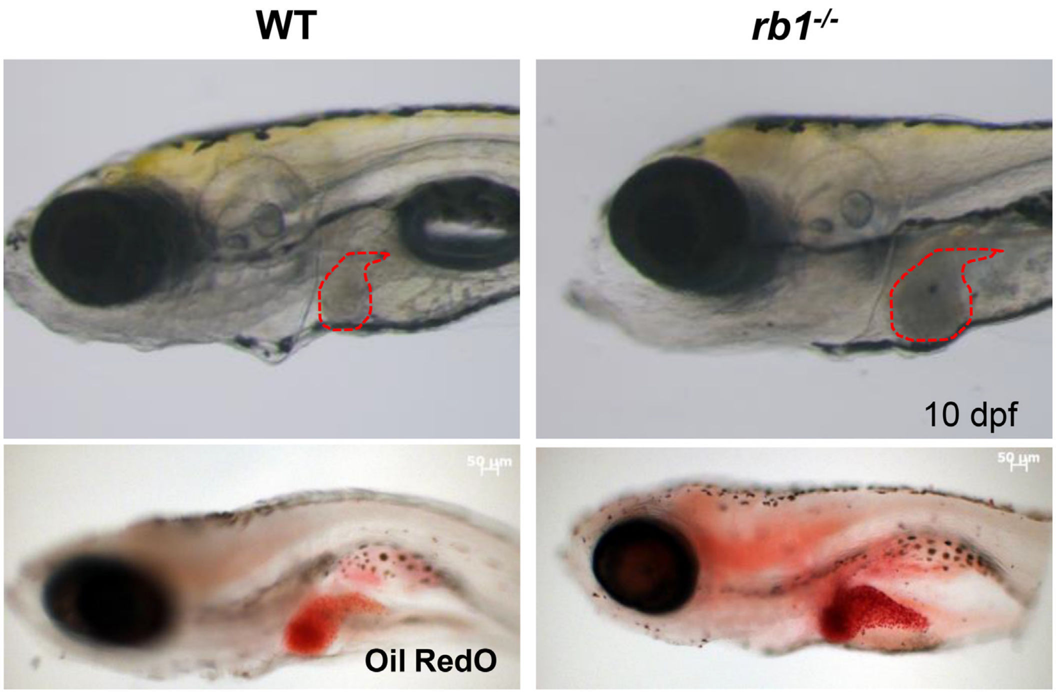

Supplementary Figure 3: TALEN-mediated mutations of *rb1* resulted in a marked enlarged liver in zebrafish larvae. Homozygous *rb1* mutant larvae were generated by incrossing heterozygous F1 zebrafish with same frameshift mutated alleles (11bp deletion). *rb1* mutants exhibited flattened swimming bladder, and enlarged liver at 7 day post fertilization (dpf) and did not survive until 10 dpf (red broken line). The increased lipid accumulation in liver of *rb1* mutants was visualized by Oil Red O staining. WT: wild type embryo, $rb1^{-/-}$: homozygous *rb1* mutant.

Supplementary Table 1: DEG of the tumors induced by *rb1* somatic inactivation. See Supplementary_Table_1

Supplementary Table 2: Gene set enrichment analysis of the tumors induced by *rb1* somatic inactivation. See Supplementary_Table_2

Supplementary Table 3: Heatmap lists of DEG among the tumors induced by *rb1* somatic inactivation, human medulloblastomas, and human PNETs. See Supplementary_Table_3
